# Supplementary material for: Identification of photoperiod-induced specific miRNAs in the adrenal glands of Sunite sheep (Ovis aries)
Source: Front Vet Sci. 2022 Jul 22;9:888207. doi: 10.3389/fvets.2022.888207 (PMC9354845; doi:10.3389/fvets.2022.888207)
Supplement: Supplementary file 1 [file Data_Sheet_1.ZIP › Supplementary file/miRDeep2 file.zip!/miRDeep2 file/Know_Structure/LP42A1/oar-mir-668.pdf]

miRBase precursor : oar-mir-668
Total read count : 22
oar-miR-668-5p read count : 1
oar-miR-668-3p read count : 21
remaining reads : 0

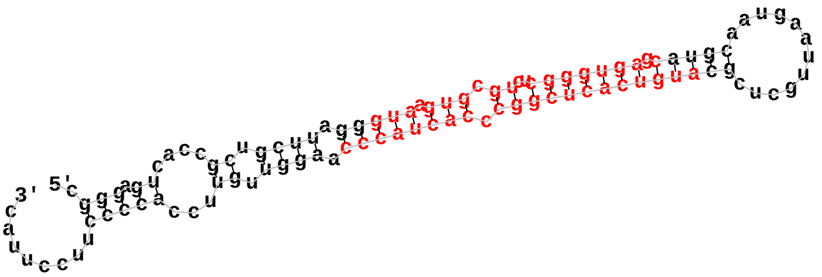

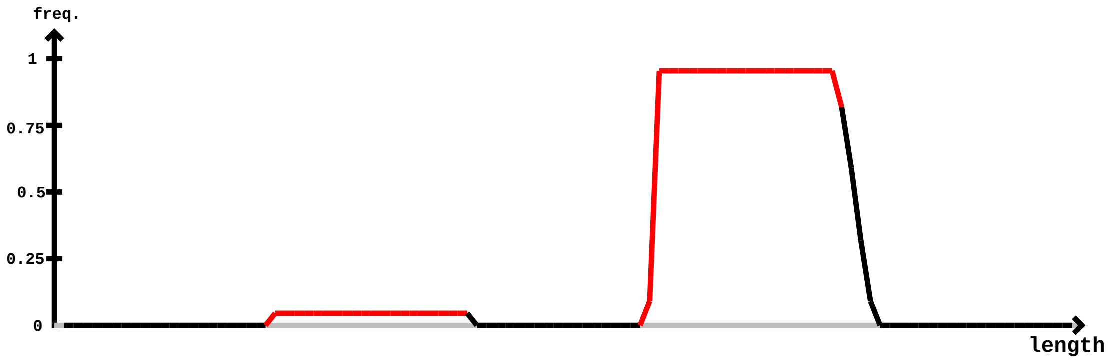

oar-miR-668-3p

oar-miR-668-5p

```
5'- cgggagucaccgcugcuuaggguaagugcgucucgggugagcaugcaaugaauugcucgcaugucacucggcccacuacccaagguuguuccaccccuuccuuac   -3'    exp
    .(((.((....((.((((.(((((.(((.((..(((((((.(((((............)))))))))))))).)))))))).)))).))...)))))........    reads    mm    sample
    .....................guaagugcgucucgggugagc...............................................................    1        0     seq
    .............................................................augucacucggcccacuacc.........................    1        0     seq
    .............................................................augucacucggcccacuaccc........................    1        0     seq
    ..............................................................ugucacucggcccacuacc.........................    2        0     seq
    ..............................................................ugucacucggcccacuaccc........................    4        0     seq
    ..............................................................ugucacucggcccacuaccca.......................    6        0     seq
    ..............................................................ugucacucggcccacuacccaa......................    5        0     seq
    ..............................................................ugucacucggcccacuacccaag.....................    2        0     seq
```
